# Genomic Analysis of Gut Bacteria in *Tecia Solanivora*: Insights into Host Functions and Their Potential in Biocontrol

**DOI:** 10.1007/s00284-026-04929-8

**Published:** 2026-06-25

**Authors:** Luisa Pantoja, Hillary Sharid Manotas-Viloria, Javier Vanegas

**Affiliations:** 1 Departamento de Biología, Pontificia Universidad Javeriana, Cra. 7 # 47a-15, Bogotá, Colombia; 2 Departamento de Ciencias de La Salud, Universidad Colegio Mayor de Cundinamarca, Cl. 28 #5B-02, Bogotá, Colombia; 3 Departamento de Biología, Universidad Antonio Nariño, Cra. 3 Este # 47a-15, Bogotá, Colombia

## Abstract

Gut bacteria in insects play critical roles in host physiology, contributing to both beneficial and harmful functions. However, the functional role of gut bacteria in *Tecia solanivora*, a major pest of potato crops in Central and South America, remains poorly understood. This study provides the first genomic and functional characterization of bacteria isolated from the gut of *T. solanivora*, focusing on their contributions to host fitness and their potential applications in pest control. Seven bacterial strains were identified: *Duffyella gerundensis* Ts1, *Enterobacter ludwigii* Ts2, *Serratia quinivorans* Ts3, *Duffyella* sp. Ts4, *Rahnella variigena* ATs1, *Rahnella variigena* ATs2, and *Stenotrophomonas* sp. ATs4. Genomic analyses revealed genes implicated in digestion and nutrient acquisition (e.g., *ribABCDE* operon for riboflavin synthesis and the *ISA* gene for isoamylase synthesis), insecticide detoxification (e.g., *nfsAB* encoding nitroreductase), and protection against natural enemies (e.g., *impABCEFGHJLM* operon for type VI secretion system). Experimental validation confirmed the activity of key functions. Notably, *E. ludwigii* Ts2 and *S. quinivorans* Ts3 exhibited entomopathogenic activity against their host insect, causing larval mortality rates of 63.3% and 40.8%, respectively. Notably, *R. variigena* ATs1 and *Rahnella variigena* ATs2 demonstrated phytopathogenic activity against potato tubers (97% damage), identifying them as opportunistic pathogens. These findings advance understanding of insect-microbe interactions, identify promising candidates for biological pest control, and highlight the importance of comprehensive safety assessments in agricultural applications.

**Graphical Abstract:**

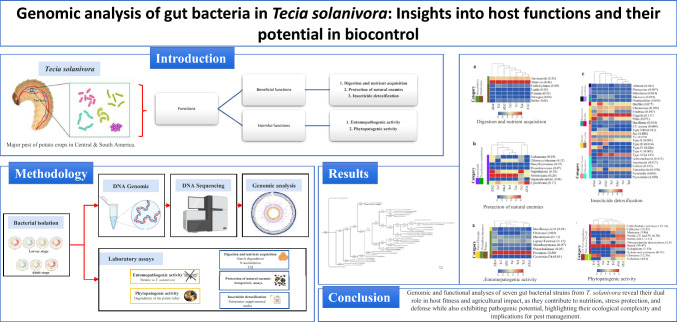

**Supplementary Information:**

The online version contains supplementary material available at 10.1007/s00284-026-04929-8.

## Highlights

- Seven bacteria were identified from larval and adult *T. solanivora* gut microbiome.
- Gut bacteria show both beneficial and harmful interactions with *T. solanivora*.
- Bacterial genes support digestion, detoxification, and defense in *T. solanivora.*
- *E. ludwigii* Ts2 and *S. quinivorans* Ts3 have entomopathogenic activity.
- *R. bonaserana* ATs1 and *Rahnella* sp. ATs2 causes potato disease symptoms.

**Supplementary Information:**

The online version contains supplementary material available at 10.1007/s00284-026-04929-8.

## Introduction

Insects represent the most diverse and abundant animals worldwide. Their evolutionary success is linked to symbiotic interactions with microorganisms, particularly those residing in the gut [1]. The insect gut microbiome comprises diverse microorganisms, with the most common bacteria. These bacteria play key roles in the host’s biology, contributing to beneficial and harmful functions that influence the host’s survival and fitness [2].

One of the primary functions of gut bacteria is enhancing nutrient acquisition. Their metabolic activities enable the breakdown of complex plant polysaccharides through the production of enzymes such as cellulases, hemicellulases, and pectinases [1]. For instance, in *Cassida rubiginosa,* symbiotic bacteria degrade pectin, allowing the insect to extract nutrients from otherwise nutritionally poor diets [3]. Moreover, gut microbiomes synthesize essential amino acids and vitamins that insects cannot produce independently. In *Plutella xylostella*, gut bacteria convert dinitrogen into ammonia, which is then used for nutrient synthesis [4]. Similarly, *Rhodococcus rhodnii* supplies B vitamins to *Rhodnius prolixus,* while *Ishikawaella capsulatus* supplies amino acids to *Megacopta punctassima,* underscoring the vital role of gut bacteria in insect survival [5].

In addition to aiding digestion and nutrient synthesis, gut bacteria help protect insects from natural enemies by producing toxins, antimicrobial peptides, and bactericidal compounds that target pathogens while preserving beneficial microbes [6]. These protective functions are critical in lepidopteran pests. For instance, *Enterococcus mundtii* in *Spodoptera littoralis* produces antimicrobial compounds that inhibit Gram-positive bacteria without disrupting the native microbiota [7]. Likewise, in other insects such as *Bombus terrestris,* gut bacteria produce toxins that suppress *Crithidia bombi,* strengthening the hosts defense [8]. These protective functions highlight the gut microbiome role in modulating host immunity and enhancing survival under pathogen pressure.

The gut microbiome plays a key role in detoxification and insecticide resistance by producing enzymes that degrade or modify toxic compounds, thereby reducing pesticide toxicity and enhancing insect survival [9]. For instance*, P. xylostella* resists chlorpyrifos due to *Enterobacter* sp. and *Serratia* sp., which metabolize the insecticide [10]. Likewise, *Stenotrophomonas* and *Enterococcus* sp. help *Bombyx mori* tolerate organophosphate insecticides [11]. This bacterial activity reduces toxicity and improves host survival, highlighting the microbiota role in insect adaptation to chemical treatments.

However, under stress conditions or gut dysbiosis, certain commensal bacteria can transition into opportunistic pathogens, leading to systemic infections that compromise host survival [2]. For example, *Pseudomonas aeruginosa*, *Alcaligenes faecalis*, and *Bacillus megaterium* become virulent in *Hylesia matabus* under physiological stress [12]. Similar transitions are observed with *Serratia marcescens* in *Rhynchites bacchus* and *Ostrinia nubilalis* [13]*,* as well as *Flavobacterium* sp. and *Klebsiella* sp. in *S. littoralis* [14]. These findings highlight the dynamic nature of host-microbiota interactions and their implications for biological control strategies.

Beyond influencing insect physiology, gut bacteria also mediate plant-pathogen interactions, adding complexity to pest management. Some insect-associated microbes exhibit phytopathogenic behavior, exploiting their symbiotic relationship with insect vectors to enhance transmission and virulence [15]. *Xylella fastidiosa*, transmitted by sap-feeding insects, blocks xylem flow, impacting citrus and grapevine crops [16]. Likewise, *Pectobacterium carotovorum* degrades plant cell walls, contributing to potato blackleg [15]. Thus, the insect microbiome not only influences host physiology but also shapes plant disease dynamics, underscoring its relevance in integrated pest management.

Traditionally, research on insect gut microbiota has relied on culture-dependent methods, which provide a limited view of microbial diversity and function [1]. Recent advances in genomic technologies have expanded this perspective, offering deeper insights into microbial roles in host biology. Despite these advancements, the gut microbiome of *T. solanivora*, a pest responsible for severe potato tuber losses ranging from 50 to 100% in Central and South America, as well as the Canary Islands, remains understudied [17]. This study addresses this gap through genomic analysis and functional validation, revealing microbial roles in host interactions and identifying candidates for biological pest control, while underscoring the need for safety assessments in agricultural applications.

## Material and Methods

### *Tecia Solanivora* Breeding, Isolation of Gut Bacteria, and Cultivation Conditions

Colonies of *T. solanivora* were maintained in the Entomology laboratory at Universidad Antonio Nariño. Adults were provided with 10% sucrose solution and water, while larvae were reared on *Solanum tuberosum* tubers. Rearing conditions were set at 19 ± 1 °C, 70 ± 5% relative humidity, and a 12/12 light/dark photoperiod [17].

Fourth-instar larvae (n = 10) and adults (n = 10) of *T. solanivora* were surface-sterilized by immersion in 70% ethanol (1 min), 5% sodium hypochlorite (3 min), and rinsed three times with sterile distilled water. After confirming sterilization, the guts were aseptically dissected in phosphate-buffered saline (PBS, pH 7.4) and homogenized in 1 ml of sterile distilled water using a mechanical homogenizer. Homogenates were serially diluted to 1 × 10⁻⁷, and 50 μL aliquots were plated on Luria–Bertani (LB) agar [2]. Following 24 h incubation at 30 °C, morphologically distinct colonies were purified, confirmed by Gram staining, and preserved in 25% glycerol at -80 °C. Seven distinct bacterial strains were obtained: four from larvae and three from adults.

### Genomic DNA Extraction, Genome Sequencing, and Assembly

Each bacterial strain was individually cultured to obtain axenic cultures, from which genomic DNA was extracted using the Wizard® Genomic DNA Extraction Kit (Promega, USA), following the manufacturer’s protocol. DNA quality was evaluated using 1% agarose gel electrophoresis, and concentration was determined with a NanoDrop® spectrophotometer (Thermo Fisher Scientific, USA). For high-throughput sequencing, genomic DNA from each strain was ligated to unique index adapters to allow sample identification during bioinformatic processing. Indexed libraries were pooled and sequenced using a multiplexing strategy on the Illumina NovaSeq 6000 platform (Illumina, Inc., USA) with paired-end reads (2 × 150 bp), achieving an average coverage of 150X per genome.

The quality of raw sequence data was assessed using FastQC v.0.12.1 [18]. Low-quality reads (Phred score < 25) and adapter sequences were removed using Trimmomatic v.0.39 [19]. The filtered reads were assembled using fullSPAdes genome assembler v3.10.0 with default parameters on the BV-BRC platform v3.28.21 [20]. Contigs were then organized at the draft genome level with ABACAS v3.7.2, in conjunction with MUMmer software [21]. Assemblies quality was evaluated with QUAST to obtain genome statistics (total length, number of contigs, GC content, largest contig, N50/L50) [20]. Additionally, genome completeness was estimated with BUSCO v5.7.1 using the bacteria_odb10 lineage dataset, which assesses the presence of conserved single-copy orthologs to determine genome completeness, fragmentation, and missing gene content [22].

### Taxonomic Analysis

Assembled draft genomes were validated using CheckM on the BV-BRC platform (v3.54.6) [20]. Species delineation methods/gene comparisons for differentiating strain from related bacterial strains were calculated using three different approaches. Average Nucleotide Identity (ANI) was calculated using ANIb from JSpeciesWS Online Service analysis platform (v5.0.3) based on BLAST + to assess species-level identity (≥ 95%) [23]. The dDDH calculated by using the Genome-to-Genome Distance Calculator (GGDC) 3.0 with Formula 2 (identities/high-scoring segment pairs length), as recommended by DSMZ for species-relatedness [24]. As a complementary method, near full-length 16S rDNA gene sequences were extracted from the assembled draft genomes using the annotation data from the RASTtk pipeline on the BV-BRC platform v3.54.6 [20]. These sequences, all greater than 1300 bp in length, were aligned against type strains with validly published names using the NCBI rRNA/ITS BLAST tool with the [type material] filter enabled [15].

Phylogenomic relationships among the strains were inferred using the Codon Tree pipeline implemented in the BV-BRC Phylogenetic Tree Service [20]. This approach constructs bacterial phylogenies from concatenated amino acid and nucleotide sequences derived from a randomly selected subset of the BV-BRC Global Protein Families (PGFams) [20]. For each selected PGFam, protein sequences were aligned using MUSCLE v3.8.31, and the corresponding coding sequences were aligned according to the protein alignment using the Codon align module implemented in BioPython v1.79 [25]. The resulting alignments were concatenated into a single PHYLIP-formatted dataset, and a partition file describing protein and codon positions (first, second, and third) was generated. Phylogenetic inference was conducted under the Maximum-Likelihood framework, using RAxML v8.2.12. Branch support was estimated with 1000 rapid bootstrap replicates.

### Genome Annotation

The functional and structural annotation of coding sequences (CDSs) was conducted using the RAST tool kit (RASTtk), integrated within the BV-BRC platform v3.54.6 [20], RASTtk utilizes curated gene annotations from the SEED database to annotate prokaryotic genomes. The tool employs tRNAscan-SE to identify tRNA genes and BLASTN to detect repeat regions, with CDS being predicted using Prodigal and Glimmer [20].

CDSs were aligned to the Kyoto Encyclopedia of Genes and Genomes (KEGG) database using BlastKOALA to assign KEGG Orthology (KO) numbers based on the highest-scoring homologs [26]. To specifically assess traits relevant to digestion and nutrient acquisition, insecticide detoxification, defense against natural enemies, and entomopathogenic or phytopathogenic activities, we employed a targeted analysis based on a curated list of 1309 genes retrieved from the literature. This gene-centric method was used to guide the identification and classification of bacterial functions relevant to host biology. These genes were grouped into predefined functional categories such as digestion and nutrient acquisition, insecticide detoxification, defense against natural enemies, and harmful functions (entomopathogenic and phytopathogenic) to structure the subsequent comparative analysis. Gene presence was determined through KO annotations obtained from BlastKOALA [27]. The presence/absence matrix was then constructed in Excel using the VLOOKUP function, which matched KO identifiers from the annotated draft genomes against the reference gene list to assign “1” (present) or “0” (absent) values. Functional profiles across strains were subsequently visualized using TBtools to produce heat maps [28].

### Laboratory Assays for Beneficial Functional Validation

Laboratory assays validate the functions identified through genomic analysis. Enzymatic activity assays determined the seven strains capacity for digestion and nutrient acquisition. Amylase production was tested on LB agar with 0.2 g potato starch, followed by iodine addition to detect starch hydrolysis [29]. Protease activity was evaluated on skim milk agar, where lysis zones indicated proteolysis [15]. Lipolytic activity was determined on LB agar with 1% Tween 20, identified by a white precipitate around colonies [9]. Nitrogen fixation was tested on NFB medium, with a green to blue color change indicating fixation [30]. Sugar metabolism was assessed using the Triple Sugar Iron (TSI) test, where fermentation changed the medium from red to yellow [29]. Insecticide susceptibility was assessed by disk diffusion, exposing bacteria to Lambda-Cyhalothrin and Profenofos insecticide concentrations (20, 50, and 100 ppm), with inhibition zones measured in Image J software [31]. Resistance, indicated by small or absent zones, suggested the ability to metabolize or neutralize insecticides [9]. Defense against natural enemies was tested against five insecticidal bacteria of *T. solanivora* (Table S1) via cross-streak, with inhibition zones measured in Image J software [31].

### Laboratory Assays for Harmful Functional Validation

A modified feeding assay was used to evaluate the entomopathogenic activity of bacterial strains against *T. solanivora* larvae. Sterilized potato slices (1.5 mm) were inoculated with bacterial suspensions (1 × 10⁷ cfu ml^−1^) and 40 first instar larvae per plate. After 72 h in darkness, larval mortality was recorded and corrected using the Abbott formula. Negative controls (potato slices with larvae and sterile distilled water) were included, and tests were performed in triplicate under standardized conditions [32]. Also, the phytopathogenic activity of the strains in inducing potato tuber rot was verified through a tuber slice assay. Potato slices were sterilized, inoculated with a 20 µL bacterial suspension (1 × 10⁷ cfu ml^−1^), and incubated at 25 °C for 48 h. Negative controls with cell-free slices were included. Tuber damage was evaluated using a predefined damage scale (Figure S1) [33]. Each treatment was replicated five times, and two independent experiments were conducted.

### Data Analysis

Data were first tested for normality and homogeneity of variances using the Shapiro–Wilk and Levene’s tests, respectively. For data meeting parametric assumptions, a one-way analysis of variance (ANOVA) was used, followed by a Tukey’s HSD test for post-hoc multiple comparisons**.** Datasets that did not meet these assumptions were analyzed using the Kruskal–Wallis test followed by Dunn’s test for pairwise comparisons. Results are expressed as mean ± standard error (SE), and differences were considered statistically significant at a p-value < 0.05.

## Results

### Isolation and Genomic Characterization of gut Bacteria From *Tecia Solanivora*

Seven distinct bacterial strains were isolated from the digestive tracts of *T. solanivora,* including four from larvae (Ts1—Ts4) and three from adults (ATs1, ATs2, ATs4). All strains exhibited Gram-negative rod morphology and formed smooth, white-to-yellow colonies on LB agar (Table S2). Draft genome sequencing of these strains yielded assemblies with an average genome size of 5.0 Mbp, distributed across 28 to 195 contigs. Sequencing depth averaged 150 × , and the G + C content ranged from 51.9% to 65.9% (Table S2). The genomic analyses also revealed key coding features, including 967 to 1321 hypothetical proteins, 2 to 5 rRNA sequences, 57 to 87 tRNA sequences, and 4335 to 5932 coding sequences (CDS) (Table S2). The BUSCO analysis showed completeness values ranging from 98.9% to 100%, with all detected genes present as single copies, fragmentation levels ≤ 0.2%, and missing BUSCOs ≤ 1.0%, reflecting the recovery of most conserved orthologs in the assemblies (Table S2).

### Phylogenetic Analysis of gut Bacteria from *Tecia Solanivora*

#### Genomic Identity Analysis

The taxonomic classification of bacterial isolates from the larvae and adults of *T. solanivora* revealed distinct affiliations. Among the larval strains, Ts1 showed genomic identity values consistent with *Duffyella gerundensis* (ANI > 90%, dDDH > 70%), while Ts2 showed similarity values consistent with *Enterobacter ludwigii* (ANI > 90%, dDDH > 70%). Ts3 demonstrated close relatedness to *Serratia quinivorans* (ANI < 90%, dDDH > 90%), whereas Ts4 represented a novel lineage within the *Duffyella/Erwinia* clade, with ANI values < 74% and dDDH below 25%. Adult-derived strains displayed distinct taxonomic patterns. ATs1 and ATs2 were related to *Rahnella* genera based on ANI (> 90%) and dDDH (> 90%) values with *R. variigena* CIP 105588 (Table S3). 

The 16S rDNA analysis showed larval isolates were identified as *D. gerundensis* (Ts1), *E. ludwigii* (Ts2), *S. quinivorans* (Ts3), and *Duffyella* sp. (Ts4), all with 100% query coverage and ≥ 99.78% identity. Adult isolates ATs1 and ATs2 were related with *R. variigena* by NCBI database revision with sequence identities of 99.84% but a query coverage below < 100% (Table S3). ATs4 was correlated with *Stenotrophomonas* species (*S. maltophilia* and *S. africana*) with sequence identities ranging from 98.36% to 99.87% and a query coverage ranging from 94 to 100% (Table S3).

#### Phylogenetic Relationships

The phylogenetic tree was rooted using representatives of the genera *Rhizobium*, *Pseudomonas*, *Vibrio*, *Citrobacter*, *Salmonella*, *Escherichia*, *Klebsiella*, and *Hafnia* as outgroups for larval isolates, and *Vibrio*, *Rhizobium*, and *Pseudomonas* for adult isolates. Bootstrap support values ranged from 84–100% across major clades.

**Fig. 1:**
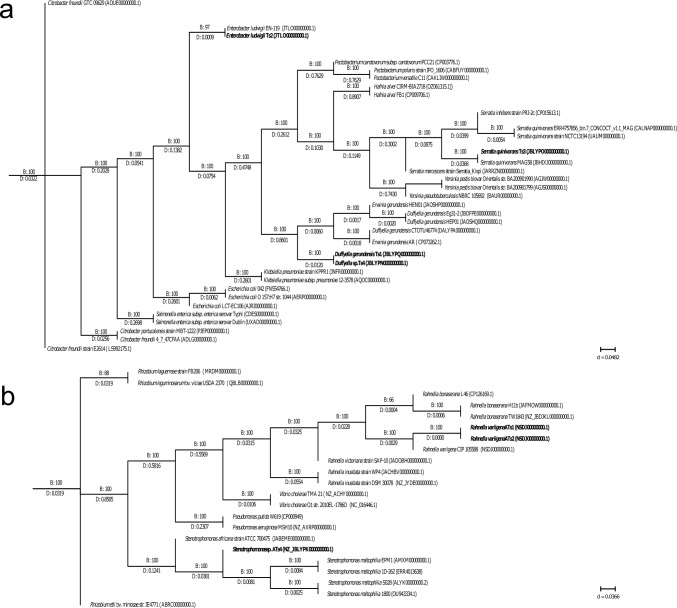
Phylogenetic tree of bacteria isolated from the gut of *T. solanivora*. (**a**) Larvae stage bacteria. (**b**) Adult stage bacteria. Maximum Likelihood (ML) phylogenetic tree showing the evolutionary position of SGTMT inferred from concatenated single-copy protein sequences. The analysis was performed using RAxML v8 under the LG substitution model (Le & Gascuel, 2008), with among-site rate heterogeneity modeled using the CAT approximation (25 rate categories). Branch lengths are proportional to the number of substitutions per site. Node values represent bootstrap support percentages obtained from 100 rapid bootstrap replicates. The scale bar indicates evolutionary distance in substitutions per site

Larval isolates formed three well-supported clades (Fig. 1A). Ts1 and Ts4 clustered together with a genetic distance of 0.0120 and showed distances of < 0.9 to other strains within the *Erwinia–Duffyella* clade, including *E. gerundensis* HEN01, *D. gerundensis* Eg31-2, and *D. gerundensis* HEP01, all supported by 100% bootstrap values. Ts2 grouped with *E. ludwigii* strain EN-119, with a genetic distance of 0.001 and 100% bootstrap support. Ts3 clustered with *S. quinivorans* MAG58, showing genetic distances of 0.03 and 100% bootstrap support. Adult isolates formed distinct clusters (Fig. 1B). ATs1 and ATs2 constituted a monophyletic clade clearly separated from other species of the genus *Rahnella*, including *R. variigena* CIP 105588, with a genetic distance of 0.002 and bootstrap support ranging from 66–100%. They were also distinct from *R. bonaserana* strains (L46, TW1843, and H11b), with genetic distances of 0.0208. ATs4 formed an independent lineage within the genus *Stenotrophomonas*, distinct from reference strains of *S. africana* ATCC 700475 (genetic distance: 0.03; 100% bootstrap support) and from *S. maltophilia* strains (EPM1, 1D-262, S028, and 18,000), with a genetic distance of 0.008 and 100% bootstrap support.

### Functional Annotation

Genome annotation of seven bacterial draft genomes from *T. solanivora* revealed metabolic genes as the predominant category, comprising 33.8—40.2% of total genes. Among these, *S. quinivorans* Ts3 exhibited the highest proportion (40.2%) and *Stenotrophomonas* sp. ATs4 showed the lowest (33.8%). Energy production genes ranked second, ranging from 11.6% in *R. variigena* ATs1 and *Rahnella variigena* ATs2 to 13.3% in *Stenotrophomonas* sp. ATs4, reflecting metabolic versatility in the host gut. Protein processing genes accounted for 9.7 to 13.1% with *Stenotrophomonas* sp. ATs4 highest and *E. ludwigii* Ts2 lowest. Cellular processes genes varied from 6.1% in *S. quinivorans* Ts3 to 9.1% in *R. variigena* ATs1 and *Rahnella variigena* ATs2. DNA processing genes, including replication and repair, represented 4.2% to 6.0%, the lowest in *S. quinivorans* Ts3 and the highest in *Duffyella* sp. Ts4 (Figure S2, Table S4).

Stress response, defense, and virulence genes constituted 7.2—9.1%, of the genome, with *D. gerundensis* Ts1 exhibiting the highest proportion among all strains. Regulation and cell signaling genes demonstrated the lowest representation across all functional categories examined, comprising only 0.5% to 1.5% of the total genes. This distribution pattern, dominated by metabolic and energy-related functions, suggests a genetic composition oriented towards the efficient utilization of nutrients within the *T. solanivora* gut environment (Figure S2, Table S4).

### Genomic and Functional Characterization of Beneficial Traits in the Seven Bacterial Strains

**Fig. 2:**
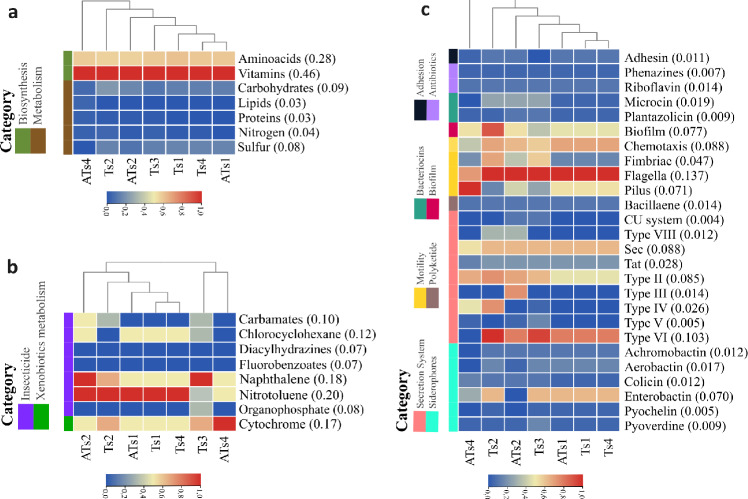
Heatmap showing the abundance of beneficial-associated genes in the gut bacteria of *T. solanivora* (**a**) Digestion and nutrient acquisition. (**b**) Insecticide detoxification. (**c**) Protection against natural enemies. The heatmap uses color coding to indicate gene presence/absence and abundance: red indicates gene presence, with intensity proportional to gene copy numbers, and blue indicates gene absence. Circles represent the count of KOs (KEGG Orthology terms) in each genome, with numbers in parentheses indicating the log2-transformed gene counts. The dendrogram at the top of the figure represents strain similarity based on the presence or absence of the genes. Abbreviations: *Duffyella gerundensis* (Ts1), *Enterobacter ludwigii* (Ts2), *Serratia quinivorans* (Ts3), *Duffyella* sp*.* (Ts4), *Rahnella variigena* (ATs1), *Rahnella variigena* (ATs2), and *Stenotrophomonas* sp. (ATs4)

Digestion and nutrient acquisition were analyzed through the genomic characterization of bacterial strains isolated from *T. solanivora,* which revealed diverse metabolic capabilities related to host nutrition (Fig. 2a, Table S5). All strains except *Stenotrophomonas* sp. ATs4 possessed genes encoding carbohydrate-degrading enzymes, particularly amylases, which correlated with positive starch hydrolysis assays (Table 1). This enzymes capacity suggests adaptation to the hosts potato-based diet. Carbohydrate metabolism patterns, assessed through the TSI test, showed that most strains demonstrated sugar fermentation capabilities, while *Duffyella* sp*.* Ts4 and *Stenotrophomonas* sp. ATs4 exhibited limited fermentative activity, indicated by alkaline/alkaline profiles (Table 1).

**Table 1:** Beneficial and harmful functions of gut-associated bacteria of *T. solanivora*

Strains								
Category	Feature	Ts1	Ts2	Ts3	Ts4	ATs1	ATs2	ATs4
Digestion and nutrient acquisition	Amylase	( +)	( +)	( +)	( +)	( +)	( +)	
	Protease	(-)	(-)	( +)	( +)	(-)	(-)	(-)
	Lipase	(-)	(-)	(-)	( ±)	( +)	( +)	(-)
	Nfb	( +)	( +)	( +)	( +)	( +)	(-)	(-)
	TSI test	A/A	A/A	A/A	K/K	K/A	A/A	A/A
		H_2_S-	H_2_S-	H_2_S-	H_2_S-	H_2_S-	H_2_S-	H_2_S-
Insecticide detoxification	Fulminator (20 ppm)	R	R	R	R	R	R	R
	Fulminator (50 ppm)	R	R	R	R	R	R	R
	Fulminator (100 ppm)	R	R	R	R	R	R	R
Protection of natural enemies (cm)	*Pseudomonas protegens* CHA0	2.63	2.10	0	0	1.19	0	0
	*Pseudomonas protegens* 59C	3.45	0.71	0.41	0	0	0	0
	*Pseudomonas* sp*.* 3B	3.64	1.40	0.35	0	0.97	0	0
	*Raoultella terrigena* C47	0.91	0	0	1.61	1.61	0	0
	*Bacillus thuringiensis* subsp. kurstaki	0.81	0	0.21	0	0	0	0
Entomopathogenic activity	Mortality of *T. solanivora* (%)	9.1	40.8	63.3	3.3	12.5	17.5	7.0
Phytopathogenic activity	Damage tuber (%)	60	80	40	10	97	97	40

Enzymatic Activity: − (no activity), + (activity); TSI reaction: A (acid), K (alkaline), H₂S Production: − (absent), + (present); Insecticide detoxification: R (resistant); Protection of natural enemies: inhibition zone; Categories marked with * indicate harmful functions. *Duffyella gerundensis* Ts1, *Enterobacter ludwigii* Ts2, *Serratia quinivorans* Ts3, *Duffyella* sp. Ts4, *Rahnella variigena* ATs1, *Rahnella variigena* ATs2, and *Stenotrophomonas* sp. ATs4.

Analysis of amino acid and vitamin biosynthesis pathways demonstrated variable distribution across strains, suggesting complementary nutritional roles within the host gut ecosystem (Fig. 2a, Table S5). A disparity was observed between genomic predictions and laboratory assays regarding lipid, protein, and nitrogen metabolism. Adult-derived strains uniquely displayed lipase activity despite similar genetic profiles across all strains. Furthermore, five strains demonstrated nitrogen fixation capacity in NFB medium, while *Rahnella variigena* ATs2 and *Stenotrophomonas* sp. ATs4 showed no growth. Although genomic analysis indicated the presence of sulfur metabolism genes, none of the strains produced detectable H_2_S under laboratory conditions. This finding illustrates a discrepancy between genetic potential and phenotypic expression (Fig. 2a, Table 1).

Insecticide detoxification was evidenced through genomic analysis of seven bacterial strains isolated from *T. solanivora,* which revealed a diverse set of insecticide-response genes. All strains except *E. ludwigii* Ts2 and *Stenotrophomonas* sp. ATs4 contained genes involved in chlorocyclohexane degradation. Three strains (*E. ludwigii* Ts2, *S. quinivorans* Ts3, and *Rahnella variigena* ATs2) harbored carbamate degradation genes, while only *S. quinivorans* Ts3 possessed organophosphate degradation genes (Fig. 2b, Table S5). Additionally, most strains contained cytochrome P450 enzymes and degradation pathways for naphthalene- and nitrotoluene-based insecticides. To validate these genomic findings, resistance to Fulminator, a commercial organophosphate insecticide, was evaluated. Remarkably, all seven bacterial strain demonstrated resistance to Fulminator, as evidenced by the absence of inhibition zones all tested concentrations (20, 50, and 100 ppm) (Table 1).

Protection against natural enemies was supported by genomic analysis that identified multiple genes associated with bacterial competition and survival. All strains harbored genes encoding siderophore production pathways and diverse secretion systems, with type II present in all strains, type VI in all except *Stenotrophomonas* sp. ATs4, and type III exclusively in *Rahnella variigena* ATs2. The analysis also revealed genes encoding various antimicrobial compounds, including bacillano-type polyketide, phenazine, riboflavin-type antibiotics, and bacteriocins, though *Stenotrophomonas* sp. ATs4 lacked microcin genes (Fig. 2c, Table S5). To assess the functional significance of these genomic features, antagonistic assays were performed against known biological control agents (Table S1). The larval strain *D. gerundensis* Ts1 showed antagonistic activity, particularly against *Pseudomonas* strains with inhibition zones ranging from 2.63 to 3.64 cm, while showing reduced activity against *Bacillus thuringiensis* subsp. kurstaki and *Raoultella terrigena* C47 (inhibition zones < 1 cm). In contrast, adult strains showed limited activity, with *R. variigena* ATs1 being the only strain to produce measurable inhibition zones against *R. terrigena* C47 (1.61 ± 0.08 cm) and *P. protegens* CHA0 (1.19 ± 0.05 cm) (Table 1).

### Genomic and Functional Characterization of Harmful Traits in The Seven Bacterial Strains

**Fig. 3:**
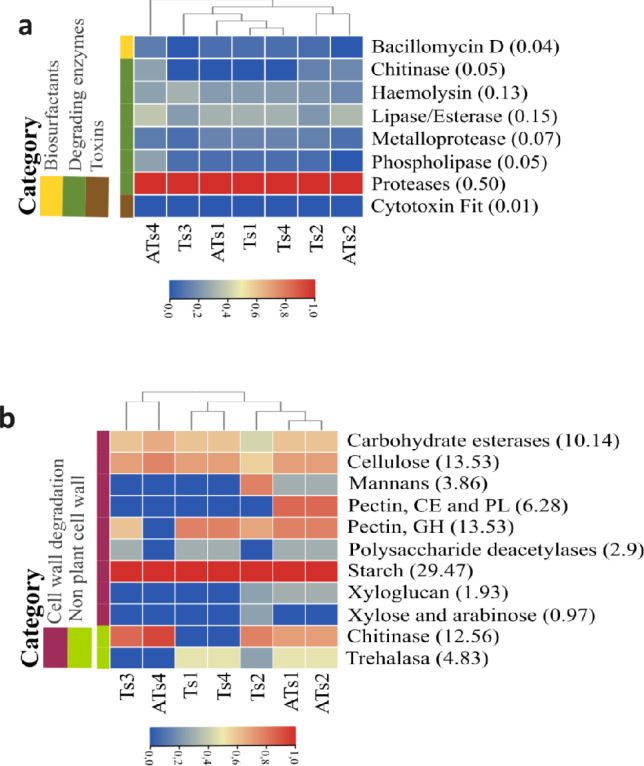
Heatmap showing the abundance of harmful-associated genes in the gut bacteria of *T. solanivora*. (**a**) Entomopathogenic activity. (**b**) Phytopathogenic activity. The heatmap uses color coding to indicate gene presence/absence and abundance: red indicates gene presence, with intensity proportional to gene copy numbers, and blue indicates gene absence. Circles represent the count of KOs (KEGG Orthology terms) in each draft genome, with numbers in parentheses indicating the log2-transformed gene counts. The dendrogram at the top of the figure represents strain similarity based on the presence or absence of the genes. Abbreviations: *Duffyella gerundensis* (Ts1), *Enterobacter ludwigii* (Ts2), *Serratia quinivorans* (Ts3), *Duffyella* sp*.* (Ts4), *Rahnella variigena* (ATs1), *Rahnella variigena* (ATs2), and *Stenotrophomonas* sp. (ATs4)

Genomic analysis revealed diverse entomopathogenic activity-associated genes in bacteria isolated from *T. solanivora* larvae and adults, including those encoding proteases, metalloproteases, lipases/esterases, and hemolysins, though *Rahnella variigena* ATs2 notably lacked phospholipase-encoding genes. Chitinase-encoding genes were restricted to *E. ludwigii* Ts2, *Stenotrophomonas* sp. ATs4, and *Rahnella variigena* ATs2, while bacillomycin biosynthesis genes were present in all strains except *Rahnella variigena* ATs2 and *S. quinivorans* Ts3 (Fig. 3a, Table S5). Additionally, *E. ludwigii* Ts2 and *S. quinivorans* Ts3 contained homologous genes related to Fit cytotoxin production, albeit in low abundance (Fig. 3a). Functional validation through laboratory bioassays demonstrated entomopathogenic activity in *E. ludwigii* Ts2 and *S. quinivorans* Ts3. Notably, *S. quinivorans* Ts3 emerged as the most potent strain, causing 63.3% larval mortality, while *E. ludwigii* Ts2 induced 40.8% mortality. These results are consistent with their potential roles as insect pathogens (Table 1).

Phytopathogenic activity was evidenced through genomic analyses that identified genes encoding enzymes for cell wall component degradation in all bacterial isolates from *T. solanivora* larvae and adults, including those targeting carbohydrates, cellulose, and starch. Pectin degradation genes were present in all strains except *Stenotrophomonas* sp. ATs4, while xylose and arabinose degradation genes were unique to *E. ludwigii* Ts2. Most strains possessed genes encoding chitinases and trehalases (Fig. 3b, Table S5). Laboratory validation of these predict enzymatic, *Stenotrophomonas* sp. ATs4, as evidenced by their ability to cause rot damage in potato tubers. Most notably, *R. variigena* ATs1 and *Rahnella variigena* ATs2 caused extensive damage, affecting 97% of the tuber surface (Table 1). These results indicate the significant potential of these strains, particularly *R. variigena* ATs1 and *Rahnella variigena* ATs2, as post-harvest potato pathogens.

## Discussion

The insect gut microbiome represents a dynamic and highly diverse ecosystem, playing key functions in host physiology. Genomic and functional analyses of seven bacterial strains from *T. solanivora* revealed roles ranging from commensal and mutualistic to pathogenicity, thereby underscoring the microbiome functional diversity and its relevance to host physiology and agricultural health.

### Genomic and Phylogenetic Characterization

Seven bacterial strains isolated from *T. solanivora* larvae and adults yielded draft genomes. The draft genomes averaged 5.0 Mbp in size, with gene content ranging from 4.335 to 5.932 CDS. Sequencing depth (150X) and G + C content variation (51.9–65.9%), completeness percentage of 99.5–100% and contamination of 0.1–0.2 supported the quality of the assemblies and revealed genomic diversity among strains (Table S2). Despite some variations in coverage, these metrics confirm the high quality of the assemblies and highlight the genomic diversity among the isolates [34].

The phylogenomic analysis of the seven strains isolated from *T. solanivora.* Strains Ts1 was identified as *D. gerundensis* showing ANI > 97% and dDDH > 70%. Ts4 was identified as *Duffyella* sp. showing ANI < 70% and dDDH < 20%. Although the Ts4 strain shows 100% identity in the 16S rRNA gene with respect to *D. gerundensis* AR, global genomic analysis refutes this classification. An ANI value of 73.12% and a dDDH of 19.50% place this strain well below the species delimitation thresholds (> 95% and > 70%, respectively) (Table S3 and Fig. 1). Other studies have reported that 16S rRNA is not sufficient for species-level resolution in Enterobacterales [35]. This detection represents the first report of *D. gerundensis* in *Lepidoptera*, suggesting an ecological adaptation of this genus beyond its established plant associations [36]. Similarly, Ts2 and Ts3 were confirmed as *E. ludwigii* (ANI > 98%, dDDH 90%) and *S. quinivorans* (ANI > 90%, dDDH 85.8%), respectively. While these species are known associates of lepidopterans like *Helicoverpa armigera*, their presence in *T. solanivora* underscores their potential roles in nutrient assimilation and carbohydrate metabolism [37, 38]. *Serratia* species are involved in pesticide detoxification in *Riptortus pedestris* [39]*.*

The adult-derived strains ATs1 and ATs2 were both taxonomically placed within *Rahnella variigena*, exhibiting an ANI of 98.6% and a dDDH value of 90% (Table S3). A strict correlation between a 70% dDDH threshold and approximately 96.6% ANI has been proposed as a robust boundary for species delimitation [34]. The values obtained for these strains are consistent with these criteria, reinforcing their classification despite the inherent taxonomic complexity of the *Rahnella* genus. Species of *Rahnella* are frequently reported in lepidopteran microbiomes and are associated with carbohydrate metabolism, particularly with the degradation of xylan [40].

In contrast, the strain ATs4 represents the most significant taxonomic finding. While showing affiliation with the genus *Stenotrophomonas* with ANI values > 90% but dDDH < 70% with species such as S. *africana* strain ATCC 700475 and *S. maltophilia* (KMM 349 and RA9) (Table S3). According to modern genomic standards, values below 95–96% ANI and below 70% dDDH are indicative of novel taxa. Therefore, ATs4 is designated as a potentially novel species within the *Stenotrophomonas* genus. Members of this genus have been shown to enhance nutrient availability and may contribute to host defense via antimicrobial volatile compounds in other insect models [41]. These results demonstrate that while some members of the *T. solanivora* microbiome are prolific and well-characterized species, others, such as ATs4, represent unique genomic lineages that warrant further functional investigation.

### Genomic Insights into Functional Potential

The phylogenetic identification of *T. solanivora* gut bacteria lays the foundation for exploring their ecological and physiological roles. Genomic analysis enables the identification of genes involved in insect-microbe interactions, offering insights into the mechanisms by which these bacteria influence insect biology. The following section discusses three key beneficial functions they may perform.

Digestion and nutrient acquisition. Genomic analysis revealed an abundance of metabolic genes, ranging from 33.8% in *Stenotrophomonas* sp. ATs4 to 40.2% in *S. quinivorans* Ts3 (Figure S2, Table S4). This aligns with previous studies on *P. xylostella* [4] and *Agrotis ipsilon* [9], which underscore the critical role of gut microbiota in metabolism, energy utilization, and nutrient absorption. Notably, the presence of genes involved in amino acid and vitamin biosynthesis suggests these bacteria play a key role in supplementing essential compounds that the hosts cannot synthesize [5] (Fig. 2a, Table S5).

Genomic analysis identified diverse enzymatic capabilities among all strains for degrading complex polysaccharides from potato plants and nectar, aiding nutrient and energy acquisition [1]. The *malQ* gene, encoding 4-α-glucanotransferase involved in starch breakdown, was present in all strains except *Stenotrophomonas* sp. ATs4 (Table S5), suggesting a symbiotic role in digestion for tuber-feeding larvae [42]. In most strains, TSI tests confirmed carbohydrate fermentation (A/A), while *Duffyella* sp*.* Ts4 and *Stenotrophomonas* sp. ATs4 showed limited activity (K/K), consistent with the absence of enzymes like alpha-amylase and beta-galactosidase (Table 1). The lack of *malQ* in *Stenotrophomonas* sp. ATs4 may reflect its association with the adult stage, which does not consume tubers.

All strains possessed key lipid metabolism genes (*pldA, glpQ, HAGH*) related to development, immunity, and metabolism (Fig. 2a, Table S5), although their precise roles in the insect remain unclear. While genes like *PLA2* are linked to immune responses [43]. *Rahnella variigena* ATs2 and *Stenotrophomonas* sp. ATs4 did not grow on lipase medium, suggesting functional divergence or incomplete pathways (Table 1). Gene variability was noted: *pldB* was absent in *Stenotrophomonas* sp. ATs4, while *plc* was exclusive to *S. quinivorans* Ts3 and *Stenotrophomonas* sp. ATs4 (Table S5). Notably, *Stenotrophomonas* sp. ATs4 carried the *HlyD* secretion gene but lacked detectable lipase activity, indicating that gene presence does not guarantee enzymatic function.

Nitrogen metabolism presents further complexity. All strains contained *gltB*, encoding glutamate synthase (GOGAT), but only five strains grew in a nitrogen-free base (NFB) medium, with *R. variigena* ATs2 and *Stenotrophomonas* sp. ATs4 being exceptions (Table S5, Table 1). While *gltB* facilitates nitrogen assimilation via the GS-GOGAT pathway, it requires an external nitrogen source and does not enable nitrogen fixation [30]. The inability of *R. variigena* ATs2 and *Stenotrophomonas* sp. ATs4 to grow in NFB medium suggests they lack the necessary machinery to convert atmospheric nitrogen into a bioavailable form, indicating adaptations to nitrogen availability within the gut of *T. solanivora* that drive metabolic specialization.

Insecticide detoxification. Genomic analysis revealed diverse insecticide-response mechanisms among strains, suggesting a potential role in host chemical defense (Fig. 2b, Table S5). The detection of cytochrome P450 enzymes in several strains suggests a robust xenobiotic metabolism capacity, as these enzymes detoxify insecticides by oxidizing lipophilic compounds into more soluble forms [44]. This pathway, widely reported in insect microbiomes, is associated with resistance to insecticides such as pyrethroids [9] and organophosphates [44].

In addition to P450 enzymes, the bacterial strains also carry genes encoding nitroreductases, which degrade nitro-based insecticides by reducing nitro groups (-NO₂) into less toxic derivatives, such as amino or hydroxylamine compounds, utilizing electrons from NAD(P)H (Fig. 2b, Table S5). This process helps mitigate the toxicity of nitro-based insecticides, commonly used in agricultural pest control, indicating that these bacteria contribute to host resistance to chemical stressors [45]. Additionally, *E. ludwigii* Ts2, *S. quinivorans* Ts3, and *Rahnella variigena* ATs2 possess carbamate kinase genes, which convert carbamates into less harmful compounds via ATP-dependent pathways [46]. This enzymatic activity is essential for counteracting the harmful effects of carbamate insecticides, which inhibit acetylcholinesterase and disrupt neural transmission. These findings align with previous research in lepidopteran pest, where gut bacteria assist in insecticide resistance through diverse mechanisms [10].

The exclusive detection of organophosphate degradation genes, including phosphotriesterase, in *S. quinivorans* Ts3 indicates a specialized capacity to hydrolyze phosphodiester bonds in organophosphate insecticides [11]. The presence of organophosphate degradation genes is relevant because organophosphates are among potato cultivation’s most commonly used insecticides. This finding highlights the potential for leveraging these bacterial strains in integrated pest management strategies while also raising concerns about the role of gut microbiota in accelerating insecticide resistance in *T. solanivora*, which could reduce the long-term efficacy of chemical control methods.

Insecticide resistance assays using Fulminator insecticide confirmed complete resistance in all bacterial strains (Table 1), suggesting synergistic interactions among the various detoxification pathways in the gut microbiota of *T. solanivora*. This supports evidence from other lepidopteran pests, where gut bacteria contribute to insecticide resistance via enzymatic degradation and metabolic modification of toxic compounds [9, 43]. Similar bacterial-mediated resistance has been documented in *P. xylostella* and *B. mori* against organophosphates [4, 10]. This diverse detoxification toolkit, likely maintained by horizontal gene transfer within the gut environment, suggests a rapid co-evolutionary response to the intense chemical pressures of modern agriculture. This raises the hypothesis that the gut microbiome acts as a key facilitator of local adaptation and the spread of resistance in pest populations.

Protection of natural enemies. The intestinal microbiota is crucial for insect immune defense, influencing host-microbe interactions and resistance to pathogens [6]. In *T. solanivora,* genomic analysis revealed competitive traits among gut bacteria, such as siderophore production, secretion systems, and antimicrobial compounds (Fig. 2c, Table S5), which may enhance host protection and modulate microbial interactions, including those with natural enemies and biocontrol agents.

Siderophores, identified in most strains, play a crucial role in iron acquisition, sequestering this essential micronutrient and limiting its availability to competing microbes, thereby restricting colonization in the iron-limited environments of the insect gut [47]. Similar mechanisms have been observed in other agricultural pests, where gut bacteria enhance host survival by outcompeting pathogens and beneficial microbes alike [48]. All strain possess Type II secretion system for releasing hydrolytic enzymes and toxins, while most also carry Type VI system that inject effectors proteins into rival bacteria, causing cell damage [49]. Additionally, *Rahnella variigena* ATs2 encodes Type III secretion system, potentially involved in modulating host physiology or symbiotic interactions [50].

Genomic analysis revealed the potential of *T. solanivora* gut bacteria to synthesize antimicrobial compounds that enhance their survival (Fig. 2c, Table S5). These include bacillano-type polyketides, which disrupt bacterial membranes or metabolic pathways, phenazines that induce oxidative stress [51], and riboflavin, which interferes with energy metabolism and redox balance, leading to cell death [49]. Bacteriocins were also identified, acting through membrane pore formation or inhibition of cell wall synthesis [7]. These mechanisms support the competitive exclusion of entomopathogenic fungi, as reported in other Lepidoptera [6, 7].

Antagonistic assays confirmed the functional relevance of the identified genomic traits, showing variable inhibitory effects against potential *T. solanivora* biocontrol agents (Table 1). *D. gerundensis* Ts1 showed the highest antagonistic activity, producing inhibitions zones of 2.63 to 3.64 cm against *Pseudomonas* strains (CHA0, 59C, and 3B), while *R. variigena* ATs1 inhibited *R. terrigena* C47 (1.61 cm) and *P. protegens* CHA0 (1.19 cm). These strain-specific interactions suggest gut bacteria may protect the host by suppressing microbial antagonist, as reported in *Helicoverpa armigera*, with *B. thuringiensis* [10]. Such interference could compromise biocontrol efficacy, making success host-dependent on microbiome composition and defensive capacity. Therefore, future biocontrol strategies may require a ‘synbiotic’ approach, combining control agents with compatible microbes or selecting agents inherently resistant to the resident microbial barrier.

These findings illustrate how the gut microbiome equips *T. solanivora* with a versatile toolkit for survival and adaptation. However, this symbiotic relationship is a double-edged sword. The same microbial community that provides these metabolic and defensive benefits also harbors a latent pathogenic potential that can be triggered against the host or exploited against its food source, revealing a complex web of ecological interactions.

### Pathogenic Potential of the Gut Microbiome in *T. Solanivora*

The gut microbiome of *T. solanivora* shows functional versatility, offering benefits to the host but also posing risks. Certain bacteria may shift toward pathogenicity or exploit the insect as a vector for plant disease transmission. The following section address their effects on insect health and potential phytopathogenic roles.

Entomopathogenic activity. The gut microbiota of *T. solanivora* displays marked functional plasticity, with some strains acting as opportunistic pathogens. Genomic analysis revealed diverse virulence-associated genes (Fig. 3a, Table S5), and functional assays confirmed insecticidal activity. Notably, strains *S. quinivorans* Ts3 and *E. ludwigii* Ts2 caused 63.3% and 40.8% larval mortality, respectively, suggesting their potential role in pathogenicity, possibly through the expression of key virulence factors (Table 1). Similar entomopathogenic traits have been observed in gut bacteria of other agricultural pests, including *H. matabus, R. bacchus* and *O. nubilalis* [12]*.*

Genomic analysis identified genes encoding proteases, lipases/esterases, and hemolysins, key mediators of insect pathogenesis (Fig. 3a, Table S5). Proteases and lipases enable tissue degradation and bacterial invasion [3], while hemolysins lyse insect hemocytes, impairing immunity and promoting systemic spread [52]. Additionally, *E. ludwigii* Ts2 harbors genes encoding chitinases, enzymes capable of degrading chitin, the main structural component of the insect exoskeleton, thereby enhancing bacterial penetration and infection [52]. Bacillomycin genes in *E. ludwigii* Ts2 suggest a competitive advantage in the gut microbiota, as this lipopeptide antibiotic disrupts microbial competitors [47]. Moreover, homologs of Fit cytotoxin genes were detected in *E. ludwigii* Ts2 and *S. quinivorans* Ts3, though at low abundance (Fig. 3a, Table S5). Fit toxins disrupt host cell membranes, causing tissue damage, promoting colonization, and immune evasion [50].

These results highlight the dual role of *T. solanivora* gut bacteria as both beneficial symbionts and potential opportunistic pathogens, raising important considerations for their use as biological control agents. Their ability to shift between mutualistic and pathogenic states underscores safety concerns in integrated pest management. Under these controlled conditions, both *E. ludwigii* Ts2 and *S. quinivorans* Ts3 reduced tuber damage caused by *T. solanivora* larvae, with *E. ludwigii* Ts2 showing the strongest protective effect (5%) (Table S6). These results provide preliminary evidence supporting their biocontrol capacity and serve as a first step toward future field validation.

Phytopathogenic activity. The phytopathogenic activity of these bacteria illustrates an ecological trade-off in the insect-microbe-plant relationship. While the insect offers dispersal and a stable gut environment, bacterial maceration of plant tissue benefits larval feeding, forming a synergistic yet destructive partnership. Genomic analyses revealed a various gene encoding enzyme for degrading major plant cell wall components, including cellulases, chitinases, and proteases (Fig. 3b, Table S5). Such enzymes, common in insect-dispersed bacteria, promote plant tissue maceration and crop damage [15]. All strains possessed cellulase genes, enabling cellulose hydrolysis into sugars [53]. Except for *Stenotrophomonas* sp. ATs4, all possessed pectin-degrading genes, encoding pectinases such as polygalacturonases, which hydrolyze pectin in the middle lamella, weakening plant tissues, facilitating microbial invasion, and accelerating decay [3].

In addition to cellulose and pectin degradation, all strains possess amylase genes, enabling starch hydrolysis into maltose and glucose (Fig. 3b, Table S5). This metabolic capacity may enhance bacterial colonization and persistence in plant tissues [40]. The combined activity of these enzymatic promotes the structural breakdown of potato tubers, increasing susceptibility to secondary infections. This enzymatic profile is consistent with findings in plant-pathogenic bacteria such as *Pectobacterium*, which are notorious for their role in soft rot diseases affecting potatoes and other crops [15].

In vitro assays confirmed the enzymatic activity predicted by genomic analyses, showing that all strains, except *Stenotrophomonas* sp. ATs4, induced rot in potato tubers (Table 1). *Rahnella variigena* ATs1 and *R. variigena* ATs2 caused the most severe damage, with 97% surface deterioration, indicating phytopathogenic potential. These results are consistent with reports on *Enterobacter* and *Serratia* species associated with post-harvest losses [52, 53]. The ability of these bacteria to colonize both insect and plant host emphasizes the need for thorough ecological evaluations prior to their use in biological control, to avoid unintended effects such as increased plant disease or microbiome disruption.

### Future Directions: From Genomic Potential to Ecological Reality

This study provides a genomic foundation for understanding the multifaceted roles of gut-associated bacteria in *T. solanivora*, encompassing functions related to digestion and nutrient acquisition, insecticide detoxification, defense against natural enemies, and entomopathogenic or phytopathogenic activities. Future research should move from genomic correlations to mechanistic validation, using gene-editing approaches (e.g., CRISPR-Cas9) to determine the contribution of key genes to these functional traits. In parallel, shotgun metagenomics of field-collected larvae will be essential to assess the ecological distribution, prevalence, and competitive dynamics of these bacteria within natural populations. Additionally, integrating host transcriptomic analyses (RNA-Seq) will clarify how *T. solanivora* responds to bacterial challenges, revealing the immune and physiological pathways that modulate these interactions. Together, these complementary approaches will bridge the gap between genomic potential and ecological function, supporting the responsible development of microbial strategies for pest management and environmental sustainability.

## Conclusion

The genomic and functional analysis of seven bacterial strains from the gut of *T. solanivora* demonstrates their dual role in host fitness and agricultural impact. These microorganisms contribute to nutrient acquisition, digestion enhancement, protection against chemical stressors, and defense from natural enemies, while certain strains exhibit pathogenic potential. The coexistence of beneficial and harmful traits, as seen in *E. ludwigii* Ts2 and *S. quinivorans* Ts3, reveals the dynamic nature of insect–microbe interactions. Such versatility poses challenges for pest management, as insecticide degradation may reduce chemical control efficacy, and antagonistic activity could hinder biological control agents. These findings expand our understanding of gut microbiota as key modulators of pest adaptation. However, further metagenomic and in situ expression studies are needed to capture the full ecological complexity of these associations. Integrating microbial ecology into pest control strategies could foster innovative, sustainable approaches that optimize crop protection while preserving agroecosystem balance.

## Supplementary Information

Below is the link to the electronic supplementary material.

Supplementary file1 (XLSX 1580 KB)

## Data Availability

These Draft Genome Shotgun projects have been deposited at DDB/ENA/GenBank under the accession: *Duffyella gerundensis* Ts1 (JBLYPQ000000000, SAMN42181656), *Enterobacter ludwigii* Ts2 (JBLYPP000000000, SAMN42181657), *Serratia quinivorans* Ts3 (JBLYPO000000000, SAMN42181658), *Duffyella* sp. Ts4 (JBLYPN000000000, SAMN42181659), *Rahnella variigena* ATs1 (JBLYPM000000000, SAMN42181660), *Rahnella variigena* ATs2 (JBLYPL000000000, SAMN42181661), and *Stenotrophomonas* sp. ATs4 (JBLYPK000000000, SAMN42181662). All strains described in this study correspond to pure and viable cultures and are fully available to the scientific community upon request. The strains are currently preserved in the institutional microbial collection at Universidad Antonio Nariño (UAN) and can be provided to qualified researchers through the corresponding author. This guarantees immediate access to the biological material while formal deposition in an international culture collection is being evaluated. All prokaryotic names reported in this study were verified using the List of Prokaryotic names with Standing in Nomenclature (LPSN; https://lpsn.dsmz.de/), accessed on February 27, 2026.

## Declarations

### Originally-Significance

The draft genomes of seven bacterial strains isolated from T. solanivora larvae and adults are analyzed to identify genes crucial for host functions and to assess their potential for use in biocontrol.
